# Mixed Neuroendocrine Carcinoma and Urothelial Carcinoma of the Upper Urinary Tract: A Case Report and Literature Review

**DOI:** 10.7759/cureus.80275

**Published:** 2025-03-08

**Authors:** Maram R Alharbi, Abdelrazak Meliti, Astabraq Alomran

**Affiliations:** 1 Pathology and Laboratory Medicine, King Faisal Specialist Hospital and Research Centre, Jeddah, SAU

**Keywords:** high-grade urothelial carcinoma, mixed carcinoma, renal pelvis, small cell carcinoma, transdifferentiation, upper urinary tract carcinoma, urothelial carcinoma

## Abstract

We describe a rare case of high-grade divergent urothelial carcinoma (UC) of the renal pelvis with neuroendocrine differentiation, specifically a small cell carcinoma (SCC) component. A 70-year-old male who presented with frank hematuria underwent a thorough clinical workup including computed tomography (CT) scan, which showed a large, contrast-enhancing obstructive right renal mass. The mass, when analyzed microscopically, showed two distinct components: high-grade urothelial carcinoma and SCC. Immunohistochemistry analysis confirmed primary dual morphological subtypes and ruled out a metastatic source. Mixed SCC and UC of the renal pelvis are extremely rare diagnoses, and staging of these tumors is difficult, highlighting the importance of integrated diagnostic approaches for an accurate characterization of complex renal tumors.

## Introduction

Urothelial cells are specialized epithelial cells lining the urethra, urinary bladder, ureter, and renal pelvis [1]. Urothelial carcinoma (UC) can be classified into lower tract urothelial carcinoma (LTUC) and upper tract urothelial carcinoma (UTUC). UTUCs of the ureter and renal pelvis are rare and represent around 5% to 10% of UCs [2]. Majority of primary UC of the renal pelvis and ureter are of high histologic grade and present at advanced stages. Additionally, the higher the grade of UC, the higher the propensity to demonstrate divergent differentiation, including neuroendocrine differentiation [3]. The coexistence of small cell carcinoma (SCC) and high-grade urothelial carcinoma (HGUC) complicates the diagnosis and management plans. Given their rarity, these tumors are often diagnosed at advanced stages, emphasizing the importance of a multidisciplinary management approach.

## Case presentation

**Figure 1:**
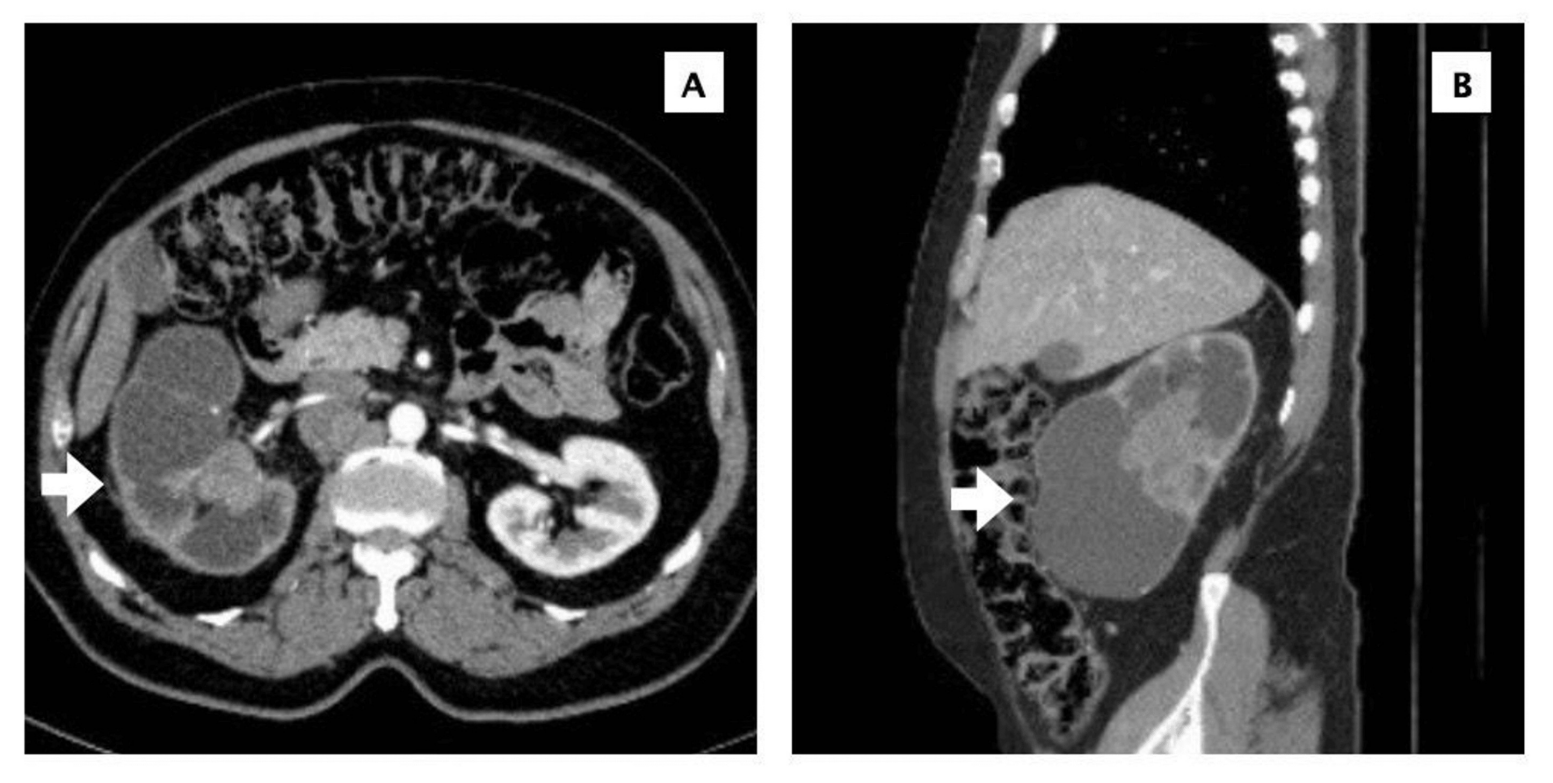
Abdominal CT, transverse view (A) and sagittal view (B), showing large right renal mass (white arrow) replacing the renal pelvis.

A 70-year-old male presented to the emergency room (ER) with right flank pain and hematuria, with no known chronic medical conditions. A computed tomography (CT) scan of the abdomen (Figure 1) revealed a large, irregular, lobulated obstructive mass in the right kidney, completely obliterating and replacing the renal pelvis. The mass measured 8.2 x 7.6 x 6.5 cm and showed no definitive vascular invasion. Additionally, there was evidence of severe chronic hydronephrosis and marked parenchymal atrophy of the right kidney.

**Figure 2:**
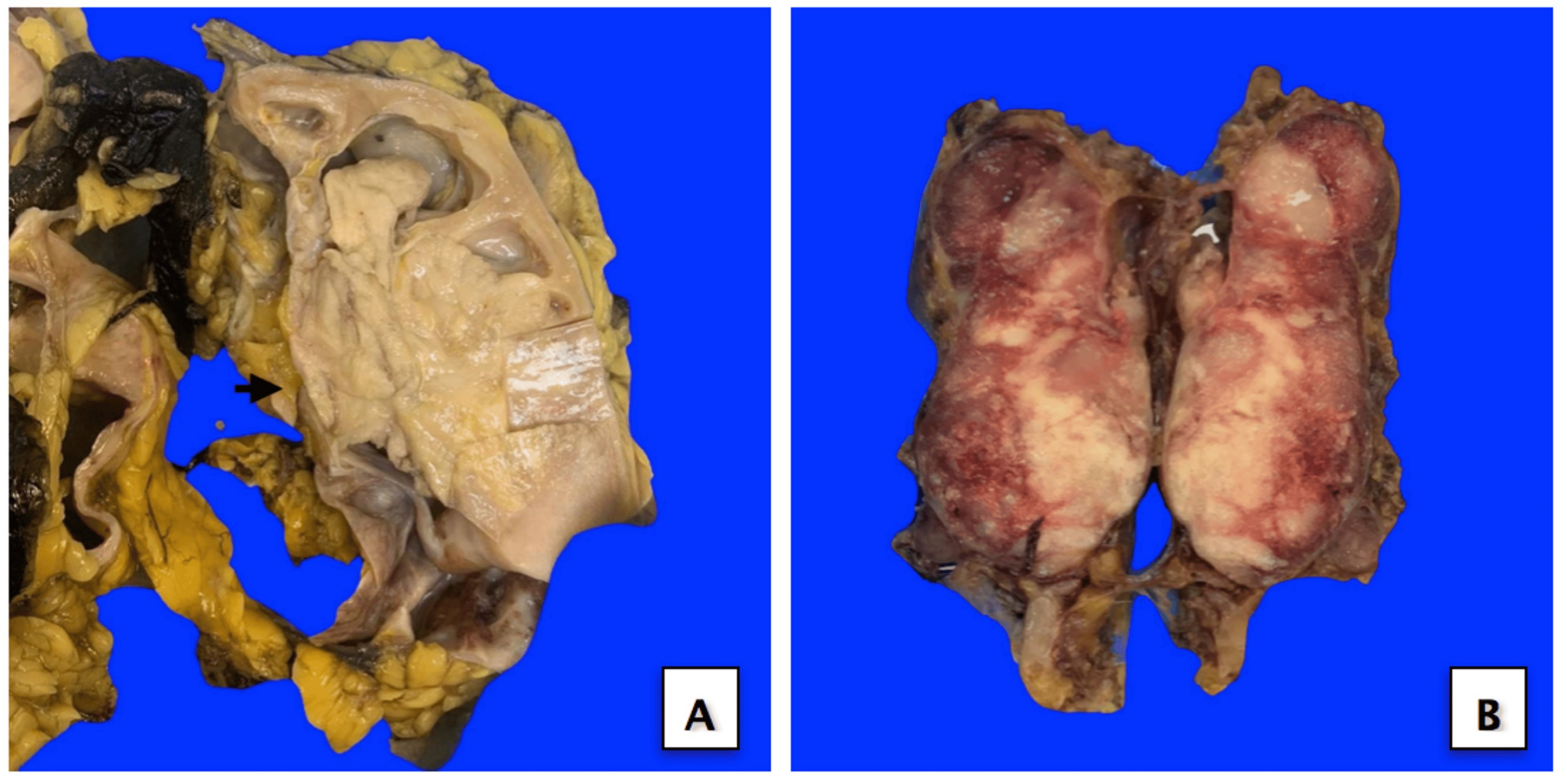
(A) Cut surface of the intra-renal pelvic mass extending into the hilum fat (black arrow). (B) A large lymph node completely replaced by tumor metastasis.

The patient underwent right radical nephroureterectomy, and the specimen was received in our pathology lab. The resected specimen measured 21 × 11 × 8 cm, consisting of a kidney proper measuring 12 × 7 × 6 cm. A well-defined, polypoid, tan-white, firm mass measuring 9.5 × 7 × 6.5 cm was identified, extending from the renal pelvis (Figure 2A). The tumor invaded the renal sinus fat and was in close proximity to the renal capsule and perinephric fat. Additionally, hilar lymph nodes (LNs) were submitted separately in a designated container. Among them, one large LN (5.9 cm in size) was entirely replaced by a metastatic tumor (Figure 2B).

**Figure 3:**
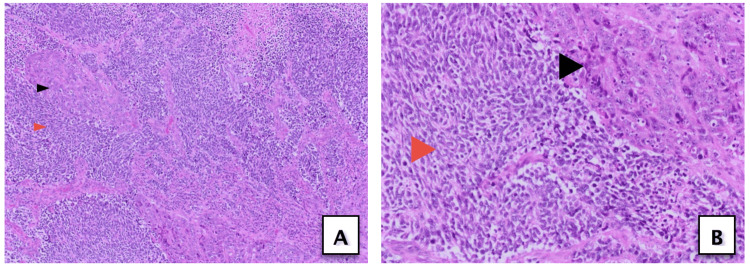
Hematoxylin and eosin stained section from the tumor showing two components including high-grade urothelial carcinoma (black arrowhead) and small cell carcinoma (red arrowhead) at (A) 10x magnification and (B) 20x magnification.

Microscopic examination showed infiltrative, high-grade, neoplastic growth with dual morphological patterns (Figure 3). The first component showed high-grade epithelioid neoplastic cells with marked pleomorphism, and the second juxta-posed component showed discohesive small hyperchromatic cells with nuclear molding, apoptosis, numerous mitotic figures, and abundant necrosis in the background.

**Figure 4:**
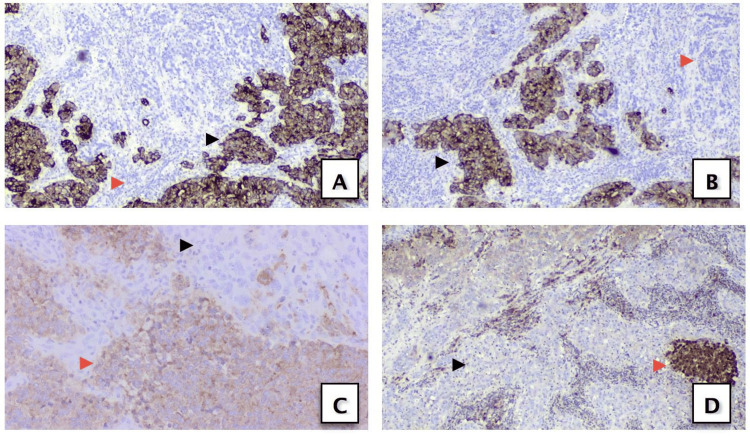
(A) CK 8/18 and (B) CK AE1/AE3 are positive in the HGUC component (black arrowhead) while negative in the SCC component (red arrowhead). (C) Synaptophysin and (D) NSE are positive in the SCC component (red arrowhead) while negative in HGUC cells (black arrowhead) (10x magnification). HGUC, high-grade urothelial carcinoma; SCC, small cell carcinoma

A panel of immunohistochemical (IHC) stains was performed, which revealed variable expression patterns (Figure 4). The epithelioid cell component was strongly and diffusely positive for CK AE1/AE3, CK 8/18, CAM 5.2, and Ber-EP4, while it was negative for GATA-3 and PAX-8. On the other hand, the small discohesive undifferentiated component showed strong and diffuse positivity for synaptophysin, CD56, and NSE, with only focal positivity for chromogranin, while it was negative for TTF-1. The above immunoprofile confirmed two tumor components and excluded a metastatic source. Both tumor components showed retained INI-1 expression.

Additionally, the large hilar LN revealed a large metastatic deposit containing both HGUC and SCC components, further indicating the aggressive nature of the tumor.

## Discussion

UC of the renal pelvis is rare and is mostly of high grade [4]. Compared to bladder UC, upper urinary tract UC tends to be more invasive and poorly differentiated, and frequently exhibits divergent differentiation [4]. UC is remarkable for its diversity of morphological appearances and can have areas of divergent differentiation, including squamous, glandular, and a small cell neuroendocrine differentiation. The latter, if present, are considered high-grade tumors [5].

The exact pathogenesis of divergent differentiation is not fully understood, and several theories have been proposed. Ordonez et al. suggested that these tumors may arise from multipotential reserve cells in the renal pelvis via transdifferentiation or dedifferentiation [6]. SCC, a subtype of neuroendocrine carcinoma (NEC), is the most common form of neuroendocrine neoplasm (NEN) affecting the urinary system, followed by large cell NEC and well-differentiated NEN [5]. SCC of the renal pelvis has a distinct feature in that they occur in association with non-neuroendocrine components, mostly in the form of divergent differentiation [3]. The transition between divergent components (HGUC to NEC) is usually abrupt. In the present case, there is a clear evidence of transdifferentiation from HGUC to SCC.

In pathological gross examination, SCC typically presents as a grayish, ill-defined mass [7]. Microscopically, the cells found are granular, and nuclei are prominent, with little cytoplasm inside the cells. Frequent mitoses and extensive necrosis are hallmark features [7].

The Current European Association of Urology (EAU) and the National Comprehensive Cancer Network (NCCN) guidelines recommend radical nephroureterectomy as the standard treatment for high-risk UTUC, while kidney-sparing surgery is advised for low-risk UTUC when feasible [8,9]. However, the aggressive nature of SCC necessitates systemic therapy. Platinum-based chemotherapy, such as cisplatin with etoposide, is commonly employed and has been shown to improve survival in cases with SCC components. Adjuvant chemotherapy is often recommended given the high risk of metastasis [10].

The prognosis for patients with HGUC with neuroendocrine differentiation is dismal, with median survival often under two years for advanced cases. Factors influencing survival include tumor stage, the proportion of the neuroendocrine component, and the extent of metastasis. In our case, the patient is still alive. During follow-up, the disease progressed despite undergoing adjuvant chemoradiation, and immunotherapy, and the patient did not show any significant response to treatment. During the course of therapy, disease progression was noted, with the development of multiple metastatic foci involving the caudate liver lobe, subdiaphragmatic region, inferior vena cava wall, cecum wall, and bladder wall, as well as bone lytic lesions suspicious for metastasis. Given the extent of disease progression and lack of response to systemic therapy, the patient was planned for palliative care.

## Conclusions

Divergent HGUC with a neuroendocrine component in the renal pelvis is a rare and aggressive malignancy requiring specialized diagnostic and therapeutic approaches. Early recognition of the dual components, particularly SCC, is critical to guide treatment. Multimodal therapies, including surgery, chemotherapy, and potentially immunotherapy, are necessary to improve outcomes. The patient’s case highlights the poor response to chemotherapy and rapid disease progression, emphasizing the need for novel treatment strategies to manage such aggressive tumors effectively. Given the rarity of mixed HGUC and SCC, our case contributes to the existing literature by reinforcing the aggressive nature of these tumors, their propensity for early metastasis, and the importance of comprehensive histopathological evaluation, including immunohistochemistry, to accurately characterize them. As these cases remain underreported, continued documentation and study are essential for optimizing treatment strategies and improving survival rates.
